# Carotenoids in Potato Tubers: A Bright Yellow Future Ahead

**DOI:** 10.3390/plants14020272

**Published:** 2025-01-18

**Authors:** Monica Sturaro

**Affiliations:** Consiglio per la Ricerca in Agricoltura e l’Analisi dell’Economia Agraria, Centro di Ricerca Cerealicoltura e Colture Industriali, via Stezzano 24, 24126 Bergamo, Italy; monica.sturaro@crea.gov.it

**Keywords:** potato, carotenoids, antioxidants, biofortification, conventional breeding, genetic engineering

## Abstract

Carotenoids, the bright yellow, orange, and red pigments of many fruits and vegetables, are essential components of the human diet as bioactive compounds not synthesized in animals. As a staple crop potato has the potential to deliver substantial amounts of these nutraceuticals despite their lower concentration in tubers compared to edible organs of other plant species. Even small gains in tuber carotenoid levels could have a significant impact on the nutritional value of potatoes. This review will focus on the current status and future perspectives of carotenoid biofortification in potato with conventional breeding and biotechnological approaches. The high biodiversity of tuber carotenoid levels and composition is presented, with an emphasis on the under-exploited native germplasm that represents a wide reservoir of useful genetic variants to breed carotenoid-rich varieties. The following section describes the structural genes involved in carotenoid metabolism and storage known to have a major impact on carotenoid accumulation in potato, together with the strategies that harnessed their expression changes to increase tuber carotenoid content. Finally, the little information available on the regulation of carotenoid metabolism and the desirable future advances in potato carotenoid biofortification are discussed.

## 1. Introduction

Potato (*Solanum* sect. *Petota*, Solanaceae) is the fifth most important food crop worldwide. There has been an increasing trend in the global production of potatoes over the past 25 years, particularly significant in the two most populous countries: India (+125%) and China (+44%), which together represent more than 40% of the total share [1]. The potato germplasm includes more than 100 species, of which only 4 domesticated, and an estimated 4000 cultivars (cvs.) grown for food, feed, and non-food uses. Around 70% of the wild species are diploid. Conversely, commercial varieties are mostly autotetraploid and comprise highly heterozygous *S. tuberosum* genotypes clonally propagated through seed tubers [2,3]. Besides energy (87 Kcal/100 g FW) and proteins of high biological value, selected potato cultivars provide substantial amounts of essential micronutrients both organic (phenolic acids, flavonoids, carotenoids, vitamins B6, B9, and C) and inorganic (K, Ca, Mg, Fe, Zn) that help meet the relative daily requirements and fight hidden hunger. Low phytate and high vitamin C levels in potato tubers ensure proper bioavailability of key minerals including Fe and Zn [4,5,6,7].

Carotenoids are terpenoid compounds produced by all photosynthetic organisms and some heterotrophic prokaryotes and fungi. In plants, they perform multiple functions essential for growth, development, and response to environmental stimuli and stress conditions. In photosynthesis, carotenoids act as light-harvesting pigments and have a key role in the assembly of the photosynthetic apparatus and its protection against photodamage. As precursors of a wide array of secondary metabolites known as apocarotenoids, including the two classes of plant hormones ABA and strigolactones, they participate in developmental and physiological processes throughout the plant life cycle. In addition, carotenoids and their derivatives contribute to the attractive colours and scents of flowers and fruits thus promoting pollination and seed dispersal [8,9,10]. Whereas carotenoid composition in photosynthetic tissues is rather constant, comprising lutein, β-carotene, neoxanthin, and violaxanthin as the main constituents [11], in non-photosynthetic organs such as fruits, flowers, and some types of stems and roots, carotenoid content and profile can be quite variable even within the same species [12].

Rising carotenoid levels in edible organs of crop plants, especially in staple crops, is desirable since these antioxidant compounds play specific roles in human health [13]. Alpha- and β-carotene and β-cryptoxanthin are the main precursors of vitamin A, whose deficiency causes impaired vision and increased susceptibility to infection and inflammation. About 500,000 children suffer annually from early blindness due to vitamin A deficiency [14]. Lutein and zeaxanthin are the main pigments of the human eye with a maximum concentration found in the macula lutea of the eye retina. By filtering blue light, they protect the eye against cataracts and age-related macular degeneration (AMD), a major cause of blindness in the elderly [15]. Due to their antioxidant activity, carotenoids have also been implicated in the prevention of oxidative stress-related diseases, including some types of cancer and dementia [16,17]. With rare exceptions [18], carotenoids cannot be produced in animals, therefore they must be assimilated regularly through the diet. Carotenoids have also a widespread use as natural colorants in the feed, food, pharmaceutical, and cosmetic industries. For instance, carotenoid-rich feed is employed to enhance the pigmentation of dairy products, egg yolk and aquaculture fish [19].

This review highlights the biodiversity of potato carotenoid content and profile and current knowledge on the genetic basis of carotenoid accumulation in tubers as a foundation for carotenoid biofortification of potatoes with conventional breeding and new biotechnological approaches.

## 2. Carotenoid Biosynthesis in Plants

**Figure 1:**
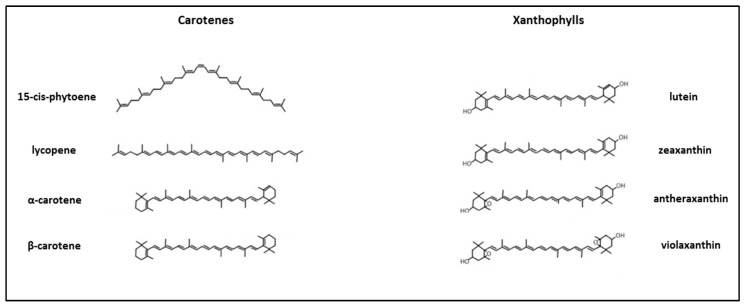
Examples of chemical structures of carotenes and xanthophylls.

Carotenoids are a vast group of over 1200 tetraterpenoid compounds comprising carotenes, linear or cyclized hydrocarbons with a variable number of conjugated double bond systems, and xanthophylls, their oxygenated derivatives [20]. Structures of some carotenoids are presented in Figure 1.

**Figure 2:**
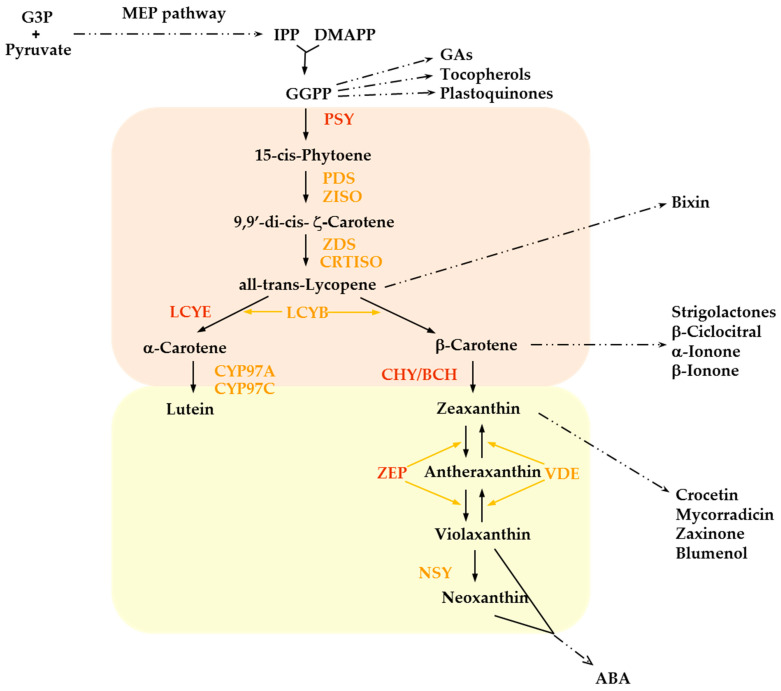
Plastidial carotenoid pathway in higher plants. Carotenes are boxed in orange, xanthophylls in yellow. Enzymes of major flux-controlling steps in potato are in dark red, the others in orange. Other GGPP-derived compounds and some of the apocarotenoids produced by carotenoid degradation are reported. G3P, glyceraldehyde 3-phosphate; MEP, metylerythritol 4-phosphate; IPP, isopentenyl diphosphate; DMAPP, dimethylallyl diphosphate; GGPP, geranylgeranyl diphosphate; GAs, gibberellins; ABA, abscisic acid; PSY, phytoene synthase; PDS, phytoene desaturase; ZISO, ζ-carotene isomerase; ZDS, ζ-carotene desaturase; CRTISO carotenoid isomerase; LYCE, lycopene ε-cyclase; LYCB, lycopene β-cyclase; CYP97A and CYP97C, cytochrome P450 carotene β- and ε-ring hydroxylases; CHY (also known as BCH), β-carotene hydroxylase; ZEP, zeaxanthin epoxidase; VDE, violaxanthin de-epoxidase; NSY, neoxanthin synthase.

Plant carotenoids are produced in all types of differentiated plastids, primarily chloroplasts in green tissues and chromoplasts in flowers, fruits, and roots. In potato tubers, amyloplasts are the main site of carotenoid biosynthesis and storage [21]. The core carotenoid biosynthetic pathway is depicted in Figure 2.

The immediate precursor of carotenoids is the C20 isoprenoid geranylgeranyl diphosphate (GGPP) produced from the condensation of isopentenyl diphosphate (IPP) and dimethylallyl diphosphate (DMPP) units, derived from the 2-C-methyl-D-erythritol 4-phosphate (MEP) pathway. GGPP acts as a substrate in other competing pathways leading to the synthesis of several secondary metabolites including gibberellins (GAs), tocopherols, plastoquinones, and chlorophyll side chains and consequently its metabolism represents a primary control point of carotenoid production. In the first committed step of carotenoid biosynthesis, two molecules of GGPP condensate to form phytoene, by means of phytoene synthase (PSY) activity. The two ensuing desaturations catalysed by phytoene desaturase (PDS) followed by an isomerization step carried out by ζ-carotene isomerase (ZISO) convert phytoene to ζ-carotene. A similar pattern of two desaturations and one isomerization follows, leading to the synthesis of all-trans lycopene. These steps involve ζ-carotene desaturase (ZDS) and carotenoid isomerase (CRTISO) activity, although in green tissues the isomerization is induced directly by light and requires chlorophyll as a sensitizer. The extended conjugated double-bond system (chromophore) of carotenoids determines their color, which can vary from yellow to orange and red, except for the colorless phytoene and phytofluene, and their photochemical features, including light harvesting and photoprotection properties.

The carotenoid pathway branches downstream all-trans lycopene, which is cyclized at both ends of the acyclic molecule following two different patterns. In the β,ε-branch, lycopene β-cyclase (LYCB) and lycopene ε-cyclase (LYCE) carry out the synthesis of α-carotene with one β-ionone and one ε-ionone end group whereas in the β,β-branch LYCB introduces two β-ionone rings to generate β-carotene, the main precursor of vitamin A. Hydroxylation of the terminal rings of α- and β-carotene leads to xanthophyll synthesis. In the β,ε-branch, cytochrome p450-type enzymes (CYP97A andCYP97C) convert α-carotene into lutein, which accumulates on plastidial membranes. In the other branch, a two-step reaction by the non-heme di-iron β-carotene hydroxylase (BCH/CHY) turns β-carotene sequentially into β-cryptoxanthin and zeaxanthin. The ensuing two-step epoxidation of zeaxanthin catalysed by zeaxanthin epoxidase (ZEP) produces antheraxanthin and violaxanthin and can be reverted by violaxanthin de-epoxidase (VDE) in the so-called xanthophyll (or violaxanthin) cycle that represents a pivotal photoprotection system. Alternatively, neoxanthin synthase (NSY) converts violaxanthin into neoxanthin: both xanthophylls are precursors of the plant hormone ABA.

The oxidative cleavage of several carotenoids, performed by carotenoid cleavage oxygenase (CCOs, also named CCDs and NCEDs in plants according to their substrate and cleavage sites), gives rise to a vast group of apocarotenoid molecules involved in various aspects of plant physiology and development. Among these are the pigments bixin and crocetin and the hormones ABA and strigolactones. In addition, CCO activity controls carotenoid homeostasis and recycling [10].

## 3. Carotenoid Content and Composition in Potato Tubers

**Figure 3:**
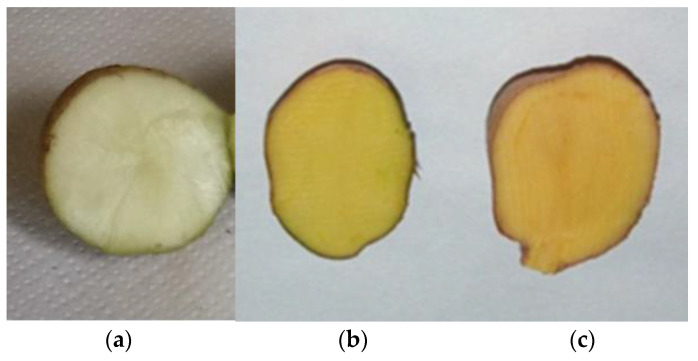
Cross-sections of potato tubers with different flesh color and from different *Solanum* species (**a**) *S. chacoense* (2n) (**b**) *S. tuberosum* (4n) (**c**) *S. phureja* (2n).

In potatoes, carotenoid level and profile closely correlate with the hue and intensity of tuber flesh color in the yellow-orange range [22,23,24,25,26,27]. The total amount of potato tuber carotenoids has been reported to vary from less than 20 to more than 2000 µg /100 g fresh weight (FW) with the highest levels found in diploid South American native cultivars of *S. phureja*, *S. stenotomum*, and *S. goniocalyx* (also indicated as *S. tuberosum* Group Phureja, Stenotomum and Goniocalyx) [5,22,24,26,28,29,30,31,32]. The xanthophylls antheraxanthin, lutein, neoxanthin, violaxanthin, and zeaxanthin, in varying proportions, constitute the bulk of tuber carotenoids in wild and cultivated potatoes, with β-carotene as a minor component (less than 3% of total carotenoid content) [22,33,34], although in some native Andean cultivars relative high levels of β-carotene were detected [25,26,28,31]. Xanthophyll esters are present in variable amounts, reaching more than 60% of total carotenoids. Their concentration positively correlates with tuber carotenoid levels, in accordance with the proposed role of esterification as a means to stabilize xanthophylls and promote their sequestration within plastids [25,28,33,35]. Carotenoid concentration and profile of diploid and tetraploid potatoes with contrasting tuber flesh color are summarized in Table 1 and examples of potato tubers with white, yellow, and orange flesh are presented in Figure 3.

White-flashed genotypes show the lowest levels of total carotenoids with a reported maximum of 450 µg /100 g FW (recalculated from [25] considering 25% dry matter) and lutein as the main constituent. In *S. phureja* accessions an inverse correlation between β-carotene and total carotenoid levels was found. The average concentration of β-carotene in white-flashed tubers exceeded that of their coloured counterparts in both relative and absolute terms [26,31]. Yellow tubers display the widest range of carotenoid concentration, with recorded levels from 14 µg/100 g FW in an old-Spanish cultivar (recalculated from [28]) to 1435 µg/100 g FW [24]. Also, the carotenoid profile is highly variable with either violaxanthin [22,26,28,33,34], lutein [24,25,26] or antheraxanthin [36,38,39] as the main component. Finally, orange-fleshed genotypes, all diploid, are carotenoid-rich, with maximum levels exceeding 2000 µg /100 g FW [23], and show a distinctive profile characterized by remarkably elevated levels of zeaxanthin (up to 90% of total carotenoids) followed by antheraxanthin or lutein [26,32,40,41].

High broad-sense heritability for carotenoid content was estimated for both total (0.96) and individual carotenoids (from 0.51 for neoxanthin to 0.93 for antheraxanthin) pointing to the genotype as the main determinant of this trait [36]. Nevertheless, according to other studies, differences in environmental conditions linked to location or year of cultivation can have a significant impact on tuber carotenoid concentration and/or profile [27,29,32,37,42,43,44,45,46]. As to the influence of the growing method on carotenoid accumulation mixed results were reported. In some cases, a positive effect of organic and biodynamic vs. conventional farming on total and individual carotenoid accumulation was observed [47,48], while in others no differences or the opposite effect emerged [27,49]. Carotenoid levels, composition, and retention in tubers are also affected by postharvest storage and thermal treatments [32,39,50,51,52]. For instance, long-term cold storage was found to either increase, decrease, or leave total carotenoid content almost unaffected [29,35,53,54]. Moreover, either pairwise or triple interactions of genotype, environmental conditions, and processing method turned out to contribute to the variability in tuber carotenoid pattern and concentration, suggesting that phenotypic stability in different environments should be considered when selecting for carotenoid-rich potato genotypes [32,36,42].

## 4. Genetic Analysis of Carotenoid Accumulation in Tubers

For the genetic dissection of carotenoid accumulation in potato tuber, genome-wide scanning and candidate gene approaches were used. Through conventional genetic analysis the Y (yellow) locus was identified as the main determinant of tuber flesh color and mapped to chromosome 3 (Chr. 3) [55] in the same region where a major QTL for flesh color and carotenoid content was later detected with bi-parental populations analysis and Genome-Wide Association Studies (GWAS). This QTL, disclosed in both diploid (Phureja) [56] and tetraploid (Tuberosum) germplasm [57,58,59], was estimated to explain 26% of the phenotypic variance for flesh colour [59]. Other minor QTLs were found scattered over the potato genome [56,57,59,60,61]. Most of them do not map to genomic regions encompassing carotenogenic genes, as is the case for tomato [62]. Candidate gene analysis for tuber carotenoid levels resulted in the identification of a few structural genes with a key role in carotenoid accumulation in potatoes, as described below. At the same time, manipulation of their expression levels led to the production of transgenic varieties showing high concentration of total and/or specific carotenoids.

### 4.1. Carotenoid Biosynthesis

#### 4.1.1. Psy

The first committed, and often rate-limiting, step in carotenoid biosynthesis is catalysed by phytoene synthase (PSY) [63,64]. Overexpression of a bacterial phytoene synthase gene in potato tubers of a low carotenogenic variety resulted in a substantial increase of total carotenoids (up to 6.3-fold, 35.50 µg/g DW) and specific components, notably β-carotene (up to 10.30 µg/g DW) and lutein (up to 11 µg/g DW). Comparable results were obtained with a carotenoid-rich variety suggesting that also in potato PSY activity represents a bottleneck in carotenogenesis [65].

In addition, a positive correlation was found between *Psy2* transcript levels and tuber carotenoid content at early stages of tuber development [35] and in *Zep* down-regulated lines [66]. In mature tubers contrasting results were reported. *Psy* expression correlated with total carotenoid concentration in three yellow-fleshed cvs. [67] but not in a group of eight tetraploid varieties with contrasting flesh color [68].

No QTL for tuber color maps to the same genomic region as either *Psy1* (SOLTU DM 03G002970) on Chr. 3, 40 Mb from the *Chy2* locus, or *Psy2* (SOLTU DM 02G020840), on Chr. 2, the genes coding for the two isoforms of potato PSY. This questions the existence of superior alleles at these loci that can be exploited in breeding programs for carotenoid biofortification of potato tubers.

#### 4.1.2. Chy2

Several investigations pointed to *Chy2* (or *Bch2*) as the putative gene corresponding to the Y locus [56,69,70]. *Chy1* and *Chy2* code for the two isoforms of potato β-carotene hydroxylase, the enzyme that converts β-carotene to zeaxanthin in the β,β-branch of the carotenoid pathway. Down-regulation of both genes in tubers led to an increase of β-carotene (up to 38-fold, 0.085 µg/g DW) and total carotenoids (up to 4.5-fold) with a concomitant decrease of zeaxanthin [71].

Conversely, high *Chy2* expression, supposedly conditioned by the presence of a specific dominant allele (allele-3 or B), is associated with yellow tuber flesh color and determines a consistent flux through the β,β-branch of the carotenoid pathway leading to the synthesis of zeaxanthin and downstream xanthophylls, the main carotenoids in yellow-fleshed tubers [38,69,72,73]. Usually, the dominant *Chy2* allele-3 is not found in white-fleshed cvs., which primarily accumulate lutein and β-carotene [56,70,74]. However, other genetic factors can override the effect of this allele on tuber colour and carotenoid accumulation given that white-fleshed clones harbouring *Chy2* allele-3 as well as yellow ones lacking it were described [75,76]. For instance, a recent analysis of potato genome structure unravelled a 5.8 Mb paracentric inversion on the long arm of Chr. 3 spanning 464 genes, among which *Chy2*. An analysis of 22 diploid accessions found a strong association between tuber flesh color, *Chy2* transcript level, and the orientation of the 5.8-Mb inversion, suggesting that this structural variation is a major determinant of tuber color through its effect on *Chy2* transcription. The occurrence of *Chy2* allele-3 in the genotypes examined was not investigated [77].

#### 4.1.3. Zep

The metabolic step following CHY activity is the stepwise conversion of zeaxanthin to antheraxanthin and violaxanthin catalysed by zeaxanthin epoxidase (ZEP). Orange-fleshed, high-carotenoid cvs., which are particularly rich in zeaxanthin, show low steady-state levels of *Zep* transcript compared to yellow and white-fleshed clones [35]. Accordingly, down-regulation of *Zep* in a low-carotenogenic variety led to a significant increase of zeaxanthin (up to 130-fold, 40 µg/g DW) but also of total tuber carotenoids (up to 5.7-fold) implying that the whole carotenoid biosynthetic pathway was affected [66].

Homozygosity for a recessive allele of this locus (*zep* allele-1) in the presence of the dominant *Chy2* allele-3 determines the naturally occurring orange phenotype. *zep* allele-1 is usually found in the diploid germplasm whereas among a panel of 230 tetraploid cvs. it only occurred in 5 varieties, always in single copy, thus explaining the absence of the orange phenotype from the known tetraploid potato germplasm [72,78].

#### 4.1.4. Lcye

LCYE catalyses the committed step in the β,ε-branch of the carotenoid pathway leading to lutein synthesis. In potato, transcript levels of *Lyce* positively correlate with lutein concentration. Antisense-mediated *Lyce* silencing in a white-fleshed variety significantly raised levels of β,β-branch carotenoids, in particular β-carotene which showed a 14-fold increase (up to 0.0436 µg/g DW) [35,79]. No correlation between specific *Lcye* allelic variants disclosed in diploid potato germplasm and tuber flesh colour was found [72]. A thorough investigation of the tetraploid germplasm could clarify whether natural genetic variation at this locus can impact carotenoid content of potato.

### 4.2. Carotenoid Degradation

#### 4.2.1. Ccd4a

In Chrysanthemum (*Chrysanthemum morifolium*) the carotenoid cleavage dioxygenase encoded by the *Ccd4a* gene negatively regulates carotenoid accumulation in flowers [80]. Similarly, the potato homologous *StCcd4a* is involved in carotenoid degradation in tubers and flower petals, as assessed in RNAi studies [81]. In stable transformants, down-regulation of *Ccd4a* led to a 5.6-fold increase of total tuber carotenoids in the most strongly affected line without altering carotenogenic gene expression. Among the xanthophylls analysed in the RNAi lines, zeaxanthin showed the largest relative variation (up to 16×), suggesting that it may represent the primary substrate of CCD4a activity, although in vitro and in vivo studies pointed to β-carotene as the most likely candidate [82]. Suppressed lines often displayed aberrant phenotypes including elongated or chain tubers with a variable degree of premature spouting, ruling out *Ccd4a* silencing as an effective means to increase carotenoid levels in tubers of commercial varieties.

In agreement with its function in carotenoid degradation, in wt potatoes *StCcd4a* shows a higher expression in mature tubers from white cultivars compared to yellow, carotenoid-rich ones [81]. A negative correlation was also found between carotenoid concentration in tubers and transcript levels of *Ccd1a*, another member of the potato CCD family [41].

#### 4.2.2. Lox

Plant lipoxygenases (LOXs) are dioxygenases that catalyse the oxidation of polyunsaturated fatty acids (PUFAs) leading to the synthesis of biologically active compounds, such as the defence-related metabolites jasmonic acid and methyl jasmonate [83]. The hydroperoxyl fatty acid intermediates produced by LOX activity can oxidate carotenoid molecules, targeting their conjugated double-bond systems [84,85]. In cereals, this co-oxidation process is responsible for β-carotene degradation during grain storage and processing [86,87].

LOX gene expression and activity were detected in potato tubers and related to the tuberization process [88,89,90]. In addition, LOX activity was found to increase in stored tubers [91]. Therefore, besides CHY2 and CCD4a, LOX might contribute to the observed turnover of β-carotene-derived xanthophylls during long-term cold storage [29,35].

### 4.3. Carotenoid Storage

In cauliflower (*Brassica oleracea*) the gain-of-function *Orange* (*Or*) mutation induces β-carotene accumulation in otherwise unpigmented tissues by triggering the differentiation of non-colored plastids into chromoplasts without affecting the expression of carotenogenic genes [92,93]. The *Or* gene is highly conserved across the plant kingdom and encodes a plastid-associated DnaJ-like protein with a cysteine-rich zinc finger domain functional in protein-protein interaction.

Overexpression of the cauliflower *Or* allele in a low-carotenoid potato variety under the control of a tuber-specific promoter resulted in a six-fold increase of total tuber carotenoids and the rise of β-carotene levels up to 5 µg/g DW from negligible amounts in the untransformed control. This implies that also in potato sink capacity constrains carotenoid accumulation. Alternatively, the carotenoid sequestering structures found in the distinctive chromoplasts of *Or*-transformed tubers may help slow down carotenoid degradation. *Or*-transformed lines also showed enhanced carotenoid accumulation and stability during long term-cold storage [94,95]. Nevertheless, the genetic background conditions the outcome of the *BoOr* allele insertion into potato. Transforming two carotenoid-rich Phureja clones with the same construct used with the white-fleshed variety resulted in a limited increase of tuber carotenoids (up to 60%), mainly due to higher xanthophylls levels, while β-carotene concentration showed only small changes [54].

A naturally occurring point mutation in the *Or* gene of melon (*Cucumis melo*), causing a single amino acid substitution, turned out to be functionally equivalent to the *Or* gain-of-function mutation of cauliflower [96]. Overexpression in Arabidopsis of the potato *Or* gene (*StOr*) or its mutagenized variant bearing the melon “golden” SNP mutation led to enhanced β-carotene accumulation and increased abiotic stress tolerance, making *StOr* a candidate target for the improvement of β-carotene accumulation and environmental stress adaptation in potato [97].

### 4.4. Additional Transgenic Strategies to Increase Tuber Carotenoid Content

#### 4.4.1. “Golden” Potato

The coordinated overexpression under a tuber-specific promoter of three bacterial genes coding for phytoene synthase (*CrtB*), phytoene desaturase (*CrtI)*, and lycopene β-cyclase (*CrtY*) was reported to induce a 20–fold increase of total tuber carotenoids with outstanding amounts of β-carotene (up to 47 µg/g DW, the highest level ever recorded in potato tubers) [98]. This “push” strategy was aimed at relieving the main bottlenecks of carotenoid biosynthesis, enabling a steady metabolic flux through the pathway. Conversely, overexpression of lycopene β-cyclase (*StLCYb*) alone only doubled β-carotene levels [99].

#### 4.4.2. Dxs

In an attempt to perturb the isoprenoid biosynthesis, a bacterial gene coding for 1-deoxy-D-xylulose 5-phosphate synthase (DXS), the first enzyme of the MEP pathway, was overexpressed in tubers of a low-carotenogenic variety. Despite the relatively high *Dxs* transcript levels in some transformed lines, carotenoid content only doubled mainly due to a 7-fold increase in phytoene concentration whereas levels of downstream carotenoids were not significantly affected [100].

#### 4.4.3. Astaxanthin

Genetic engineering of potato tubers was also employed to produce the valuable ketocarotenoid astaxanthin, which is not naturally synthetized in potatoes. In one study, the *crtO* ketolase gene from the cyanobacterium *Synechocystis* was overexpressed in a transgenic potato line high in zeaxanthin. Ketocarotenoids accumulated up to 10–12% of total carotenoids in both leaves and tubers. However, astaxanthin only accounted for 4.8% of the total carotenoid content (1.8 µg/g DW) [101]. Better results were obtained by transforming a high carotenogenic *S. phureja* clone with an algal β-carotene ketolase gene (*bkt1*): astaxanthin levels in tubers reached 14 µg/g DW (47% of total carotenoids) [102]. A further increase in astaxanthin levels was achieved by stacking in a selected *S. phureja* background the cauliflower *Or* allele and two bacterial genes coding for β-carotene hydroxylase (*crtZ*) and β-carotene ketolase (*crtW*). Compared to previous attempts with the *bkt1* gene alone, a six-fold higher tuber astaxanthin content was obtained (up to 77 μg/g DW, a level considered nutritionally significant [54]).

## 5. Genome Editing

Until now, genome editing technologies have not been harnessed to improve tuber carotenoid concentration and composition because of the possible systemic side-effects of knocking out carotenogenesis genes, primarily on photosynthesis. Indeed, CRISPR-Cas9 silencing of the potato *Pds* gene led to albino mutants devoid of both carotenoids and chlorophylls, whose synthesis is coordinated [103]. In contrast, CRISPR/Cas9-based editing of the banana (*Musa* spp.) *Lcye* gene caused a drastic reduction of lutein, the most abundant carotenoid in chloroplasts, but with no or little effects on photosynthesis. Only a slight decrease in chlorophyll a/b ratio and reduced growth of fruit bunches were observed in the most affected knockout line grown under controlled conditions [104]. It should be noted, however, that in higher plants lutein is required for effective photoprotection only under strong light and the Arabidopsis lutein-deficient mutant *lut2*, impaired in LCYE activity, is viable under non-stress conditions. Likely, the observed increase in β,β-carotenoids, particularly those involved in the xanthophyll cycle (zeaxanthin, antheraxanthin, and violaxanthin) functionally compensates for lutein deficiency [105,106].

To avoid systemic side effects, genome editing could be used to target single components of multi-gene families displaying different expression patterns. For instance, the potato paralogue genes *Chy1* and *Chy2* encode the main β-carotene hydroxylase isoform of leaves and tubers respectively [71]. Alternatively, especially with polyploid genotypes, the production of heterozygous mutants with the proper balance of wt and edited alleles may lead to a modulation of the knockout effect on plant phenotype. Genome editing could also be harnessed to replicate natural mutations with a proven effect on carotenoid accumulation, such as the *Or* mutation of melon, which boosts carotenoid storage without affecting photosynthesis. Another example is the *zep-1* leaky mutation of potato, almost completely restricted to the diploid Phureja germplasm, which could be functionally reproduced in tetraploid commercial varieties to enhance tuber zeaxanthin content.

## 6. Regulation of Carotenoid Metabolism in Potato

Genetic manipulation of carotenoid accumulation in tubers has relied so far on a basic knowledge of the structural genes involved in carotenoid synthesis, storage, and turnover. Understanding the regulation of carotenoid metabolism is required to further extend the qualitative and quantitative improvement of tuber carotenoid content through biotechnological approaches. Carotenoid metabolism in plants is subjected to a complex regulation. Although some of its aspects have been elucidated, the whole picture is still missing [107,108,109]. Compared to other species like Arabidopsis and tomato, limited information is available on the regulation of potato carotenoid accumulation, a process which is likely to present distinctive features. Potato tubers are peculiar organs that have few equivalents in other cultivated or model plants [110] and the carotenoids they store, mostly xanthophylls, differ from those found in carotenoid-rich organs of closely related species such as tomato (*Solanum lycopersicum*, lycopene and β-carotene [111]) and pepper (*Capsicum annuum*, capsanthin, lutein and β-carotene [112]). Moreover, the underground growth habit of potato tubers rules out a direct role of light in the regulation of carotenoid metabolism, whereas in fruits, flowers, and photosynthetic organs light is a major cue [113,114]. As described above, metabolic engineering has shown that some structural genes are key in determining the amount and profile of tuber carotenoids and represent possible flux-controlling steps of natural carotenoid homeostasis. On the other hand, since most of the QTL identified so far underlying carotenoid accumulation in potato tubers are not linked to carotenogenic genes, a complex regulatory network is anticipated to control this metabolic process [73].

### 6.1. Transcriptional Regulation

Analysis of carotenoid gene expression has given limited information on the transcriptional regulation of carotenoid levels in tubers. In many species PSY activity, as the committed step of carotenoid biosynthesis, is tightly regulated transcriptionally and post-transcriptionally. For example, during photomorphogenesis, a light-dependent pathway involving phytochrome and cryptochrome photoreceptors modulates *Psy* expression through transcriptional activators/repressors that bind G-box motifs in the *Psy* promoter [64]. In potato, higher *Psy* transcript levels were found to correlate with higher carotenoid content in the early stages of tuberization [35] but no transcriptional regulator was shown to directly control *Psy* expression in response to developmental or environmental factors.

*Chy2* is believed to play a more significant role in tuber carotenoid accumulation, and its mRNA steady-state levels correlate with carotenoid concentration in this storage organ [38,41]. A sugar-responsive cis-element in the promoter of the dominant *Chy2* allele-3 together with relative high concentration of tuber glucose and sucrose was suggested to explain the increased transcription of this allele in a carotenoid-rich cultivar [41]. However, a subsequent study did not find any significant correlation between the levels of total or individual soluble sugars and the amount of tuber carotenoids [56]. The putative effect of the Chr. 3 genomic inversion on *Chy2* expression has not been analysed in detail.

A transcriptional regulation was suggested also for the main carotenoid degradative gene of potato tubers, *CCcd4a*, given the negative relationship between its mRNA level and carotenoid concentration in tubers with contrasting flesh colours [81]. Functional analysis of the *Ccd4a* promoter has not been reported.

### 6.2. Post-Transcriptional Regulation

Additional structural genes with a putative role in carotenoid regulation in tubers are *Zep* and *Or*. Besides the expected increase in zeaxanthin levels, downregulation of *Zep* stimulates the whole carotenoid biosynthesis suggesting a broader role for this gene in controlling carotenoid accumulation in potato tubers [66]. Accordingly, an inverse correlation between *Zep* transcript level and total carotenoid accumulation was observed in a range of potato germplasm [35]. Similarly, the tomato *high-pigment 3* (*hp3*) mutant impaired in ZEP activity shows higher carotenoid accumulation in fruit and leaves associated with enlargement of the plastid compartment size [115] and the *zep* mutation in the *aba1-1* mutant of Arabidopsis conditions a 60% higher carotenoid content in leaves [116]. It is not known whether *Zep* silencing in potato determines an increased sink capacity for carotenoid accumulation, as is the case for the tomato *hp3* mutant. The observed enhancement of *Psy* transcript levels in potato *Zep*-suppressed lines could be the main determinant of carotenoid overproduction. The natural recessive *zep-1* mutation found in native orange fleshed high-carotenoid germplasm is due to the insertion of a non LTR-retrotransposon-like sequence in the first intron which may affect mRNA processing [38,72].

In addition to regulate plastid differentiation into chromoplasts, the OR protein was shown to control post-transcriptionally PSY level and activity in Arabidopsis by directly interacting with PSY in plastids [117]. In transgenic potato overexpressing the *Or* gene higher stability of PSY was observed pointing to a similar OR-dependent mechanism of PSY regulation promoting carotenoid biosynthesis [95].

## 7. Discussion

Although French fries’ consumption may be associated with an increased risk of obesity and type 2 diabetes [118], potatoes can help meet daily caloric requirements and food security in countries where undernutrition is a concern [119]. Moreover, they provide a variety of health-promoting micronutrients including various antioxidant metabolites like the tuber colouring pigments anthocyanins and carotenoids [7,120,121]. A negative correlation between tuber carotenoid and anthocyanin content was reported in native South American potato germplasm [30]. Nevertheless, pigmented varieties high in both types of antioxidants were described, suggesting that there is no reciprocal interference in the accumulation of these two classes of nutraceuticals [37,39].

Even though metabolic engineering of potato has been successfully employed to increase both total and specific tuber carotenoids, regulatory constraints and consumers’ diffidence towards GM food crops may limit the actual marketability of the engineered varieties [122,123,124]. On the other hand, NPBTs have been effectively used for site-specific genome editing of agronomic and quality traits in tetraploid potato cultivars. Transient CRISPR-Cas9 expression or CRISPR-Cas9 ribonucleoprotein delivery into protoplasts has proven useful to regenerate edited clones devoid of foreign DNA [125].

The improvement of commercial potato varieties with conventional breeding approaches is hampered by their tetraploid nature, self-incompatibility, and high heterozygosity typical of an inbreeding-sensitive species. In addition, the widespread occurrence of toxic steroidal glycoalkaloids (SGA), primarily α-solanine and α-chaconine, among wild potato genotypes is a detrimental trait that must be counter-selected in breeding programs. Nevertheless, the genetic potential of wild potato germplasm has been successfully exploited to introgress resistance and quality genes into economically important cultivars, overcoming hybridization barriers based on differences in ploidy [3,126,127,128]. Potato breeding to enhance tuber carotenoid content can harness the high heritability of the trait and the wide genetic variation in carotenoid level and composition of the native germplasm [4,5,30,77]. For instance, the tetraploid variety Nagasaki Kogane with high levels of tuber carotenoids (845 µg/100g FW, mainly zeaxanthin and lutein) has a diploid, carotenoid-rich *S. phureja* genotype in its ancestry [129].

A recent breakthrough in the context of genomics-assisted breeding of potatoes is the sequencing of a few modern cultivars which has shed light on the complex structural and functional organization of their highly heterozygous autotetraploid genome [130,131,132]. The knowledge drawn from these sequencing efforts will ease the construction of homozygous potato genotypes devoid of the deleterious alleles linked to inbreeding depression and of the large genomic rearrangements underlying regions of extensive linkage disequilibrium. Indeed, ongoing efforts to redesign potato as a diploid, seed-propagated crop have the potential to overcome major drawbacks in potato breeding, easing the introgression of favourable genes into elite germplasm and opening the way to hybrid production [133,134,135].

## 8. Conclusions

Exploiting new plant breeding techniques (NPBTs), in particular genome editing, represents the next step in carotenoid biofortification of potatoes. Conventional genetic manipulations have already disclosed some major structural genes controlling the metabolic flux through the carotenoid pathway, making them primary targets of editing approaches. For instance, genome editing represents a potential tool to boost tuber β-carotene levels in a transgene-free background through the introduction of the gain-of-function *Or* mutation into the potato genome, provided that the proper genotypes are targeted. Genome editing and advances in genomics-assisted breeding, together with a deeper understanding of the regulatory mechanisms underlying carotenoid accumulation in tubers, may open a new era in the field of potato carotenoid biofortification, and further improve the nutritional value of this staple crop.

## Figures and Tables

**Table 1:** Carotenoid content and profile in potato tubers with different flesh color.

Taxonomic Group (Ploidy)	Flesh Color (n)	TCC	Major Components ^1^	Minor Components ^1^	Refs.
*S. tuberosum* (4n)	W (3) Y (6) DY (4)	27–74 61–157 171–343 µg/100 g FW	Lute, Viola Lute-ep. Viola, Lute, Lute-ep., Neo Viola, Lute, Lute-ep., Neo	Neo, β-Car	[22]
*S. tuberosum* (4n) *S. phureja x S. stenotomum* (2n)	W (2) Y (11)	64 and 136 111–1435 µg/100 g FW	Lute, Viola, Lute-ep., Neo Lute-ep., Viola, Lute	Zea Neo, Zea	[24]
*S. tuberosum* (4n)	W (4) Y (4)	38–62 58–175 µg/100 g FW	All: Viola, Anthe, Lute, Zea in different ratios	All: Neo, β-Cripto, β-Car	[33]
*S. tuberosum* (4n) *S. phureja x S. stenotomum* (2n)	W (7) Y (11) O (1)	38–265 107–260 878 µg/100 g FW	Lute, Viola Viola, Lute Zea, Anthe, Viola, Lut	Anthe Neo, Anthe β-Cripto, β-Car	[34]
*S. tuberosum* ssp. *andigena* (4n) *S. ajanhuiri* (2n) *S. juzpeczukii* (3n) *S. phureja* (2n) *S. stenotomum* (2n)	W (6) C (9) Y (7) P (1)	1.78–17.90 5.35–18.28 18.85–54.78 16.35 µg/g DW	All: Lute, Neo, Viola, Zea in different ratios	All: β-Car, Anthe, β-Cripto	[25]
*S. phureja* (2n)	C/LY (9) LY/Y (8) Y/DY (6)	97–262 682–1270 1258–1840 µg/100 g FW	Lute, Viola, Anthe, β-Car Viola, Anthe, Lute, Zea Zea, Anthe	Zea β-Car Lute, Viola, β-Car	[26]
*S. tuberosum* (4n)	W (3) Y (6)	101–145 218–511 µg/100 g FW	Lute, Anthe, Viola Anthe, Viola, Zea, Neo	Zea, Neo Lute	[36]
*S. tuberosum* (4n) *S. tuberosum* ssp. *andigena* (4n) *S. phureja* (2n) *S. stenotomum* (2n) *S. goniocalix* (2n)	W-P (1) LY (8) Y (46) P-W (5)	1.66 0.77–6.33 0.54–15.51 0.84–3.27 µg/g DW	Lute, Neo Lute, Neo or Viola, Neo Viola, Neo Lute, Neo, Viola	All: Anthe, β-Car, β-Cripto	[28]
*S. tuberosum* (4n) *S. phureja* (2n)	W (2) Y (3) R/P (12)	All: from 0.779 (W) to 13.3 (Y) µg/g DW	All: Lute	All: Viola, Neo, Zea, β-Car	[37]
*S. tuberosum* (4n) *S. chacoense* (2n) *S. phureja* (2n)	W (2) Y (5) O (3)	1.37 and 4.1 13.83–32.12 17.42–26.89 µg/g DW	Lute, Anthe, Neo and Viola, Lute Anthe, Viola, Lute, Zea Zea, Anthe, Lute	β-Car, Zea Neo, β-Car Viola, Neo, β-Car	[38]

^1^ = in decreasing order. TCC = Total Carotenoid Content, W = White, Y = Yellow, DY = Dark Yellow, O = Orange, C = Cream, P = Purple, R = Red, FW = fresh weight, DW = dry weight, Lute = Lutein, Viola = Violaxanthin, Lute-ep = Lutein-5,6-epoxide, Neo = Neoxanthin, Anthe = Antheraxanthin, Zea = Zeaxanthin, β-Car = β-Carotene, β-Cripto = β-Criptoxanthin.

## Data Availability

No new data were created or analyzed in this study. Data sharing is not applicable to this article.
